# Connecting genetics and gene expression data for target prioritisation and drug repositioning

**DOI:** 10.1186/s13040-018-0171-y

**Published:** 2018-05-31

**Authors:** Enrico Ferrero, Pankaj Agarwal

**Affiliations:** 1 Computational Biology, Target Sciences, GSK, Gunnels Wood Road, Stevenage, SG1 2NY UK; 2 Computational Biology, Target Sciences, GSK, 1250 S. Collegeville Road, UP12-100, Collegeville, PA 19426-0989 USA; 3 Present Address: Autoimmunity, Transplantation and Inflammation, Novartis Institutes for Biomedical Research, Fabrikstrasse 2, Basel, 4056 Switzerland

**Keywords:** Drug discovery, Drug repositioning, Genomics, Transcriptomics

## Abstract

Developing new drugs continues to be a highly inefficient and costly business. By repurposing an existing compound for a different indication, drug repositioning offers an attractive alternative to traditional drug discovery. Most of these approaches work by matching transcriptional disease signatures to anti-correlated gene expression profiles of drug perturbations. Genome-wide association studies (GWASs) are of great interest to researchers in the pharmaceutical industry because drug programmes with supporting genetic evidence are more likely to successfully progress through the drug discovery pipeline.

Here, we present a systematic approach to generate drug repositioning hypothesis based on disease genetics by mining public repositories of GWAS data and drug transcriptomic profiles. We find that genes genetically associated with a certain disease are more likely to be differentially expressed in the same disease (*p*-value = 1.54e-17 and AUC = 0.75) and that, in existing drug – disease combinations, genes significantly up- or down-regulated after drug treatment are enriched for genes genetically associated with that disease (*p*-value = 1.1e-79 and AUC = 0.64). Finally, we use this framework to generate and rank novel GWAS-driven drug repositioning predictions.

**Electronic supplementary material:**

The online version of this article (10.1186/s13040-018-0171-y) contains supplementary material, which is available to authorized users.

## Introduction

The discovery, development and commercialisation of a new drug is a long, expensive and often failure-prone process [1–3]. Drug repositioning can be a time- and cost-effective alternative where existing compounds are repurposed for diseases different from the original indication [4, 5]. These approaches can be subdivided into multiple classes, though a majority of recent computational work has focussed on two: drug-based, relying on chemical structure similarity and predictions of drug – target interactions, and disease-based, where transcriptomic readouts of disease samples and drug perturbations are combined [6].

The latter was popularised by the Connectivity Map [7, 8], an in silico pipeline to reverse-match transcriptional disease signatures with gene expression profiles obtained by perturbing cellular systems with a large panel of compounds. The Library of Integrated Network-based Cellular Signatures (LINCS) [9] project greatly expanded the pool of compound profiles, triggering further development of computational methods for drug repositioning [10, 11] as well as approaches for the validation of these in silico predictions [12].

Selecting the right targets is a key decision early in the drug discovery pipeline [13]: a large proportion of the efficacy failures in clinical programmes are due to lack of a clear link between the therapeutic target and the disease under investigation [14]. There is growing recognition that supporting genetic evidence from genome-wide association studies (GWASs) or phenome-wide association studies (PheWASs) linking target and disease can significantly increase the chances of success of drug discovery programmes [15, 16]. The large number of GWASs conducted over the past decade have delivered insights into the causal links of several diseases [17] and more and more genes are expected to be implicated in disease as the size of these studies grow, even though not all associations might be as meaningful as previously thought [18].

GWASs [19], PheWASs [20], Connectivity Map approaches [21–23] and Open Targets [24] have all been used to repurpose drugs. Here, we combine disease data from GWASs with drug perturbation transcriptional profiles and a Connectivity Map-inspired method to generate repositioning hypotheses that, unlike those in standard expression-based repurposing workflows, are supported by genetics evidence.

## Methods

### Software and code

R 3.4.0 [25] was used for all data processing and analysis. All code was versioned using Git and is hosted at https://github.com/enricoferrero/GCMap.

### Data sources

STOPGAP [26] is a database containing associations between DNA mutations occurring in diseases and likely target genes. This includes rare disease associations as well as data from GWASs. For single nucleotide polymorphisms (SNPs) in regulatory regions, associations to target gene are performed on the basis of supporting evidence including eQTL and regulatory genomics data. The complete dataset (294,505 associations between 20,015 genes and 1746 medical terms) was downloaded from https://github.com/StatGenPRD/STOPGAP/blob/master/STOPGAP_data/stopgap.gene.mesh.RData. Open Targets [27] maps diseases to relevant genes using a number of evidence types, including genes differentially expressed in the disease, germline and somatic mutations, curated pathway, animal model and literature data as well as known drugs approved for the treatment of the disease. The Open Targets API at http://api.opentargets.io/v3/platform/docs was accessed on 20th June 2017 and used to download lists of genes differentially expressed in disease (216,942 associations between 22,190 genes and 148 diseases) and links between 595 diseases and 1555 approved drugs (7351 associations). The LINCS [9] L1000 data consists of gene expression profiles obtained by perturbing different cell lines with a large collection of compounds. To obtain the complete genome-wide dataset in a convenient format, we used the Harmonizome [28], a large collection of uniformly processed biological datasets. The file downloaded from http://amp.pharm.mssm.edu/static/hdfs/harmonizome/data/lincscmapchemical/gene_attribute_edges.txt.gz contained 4,189,677 associations between 3924 compounds and 8347 genes differentially expressed after treatment, with a median of 257 genes changing for each compound.

### Data processing

STOPGAP data: gene – disease associations from rare disease sources (OMIM and Orphanet) were excluded. To ensure compatibility with other resources, gene symbols were mapped to Ensembl gene IDs with the EnsDb.Hsapiens.v75 package [29]. MeSH terms were mapped to terms in the Experimental Factor Ontology (EFO) [30] using Zooma [31]. LINCS L1000 data from Harmonizome: Entrez gene IDs were mapped to Ensembl gene IDs. Compound IDs were mapped to ChEMBL IDs with UniChem [32], using PubChem IDs as an intermediate.

### Data analysis

EFO IDs and ChEMBL IDs were used to match diseases and drugs across different resources, respectively. A Fisher’s exact test [33] was used to perform enrichment tests between gene sets and to generate repositioning hypotheses. Results were corrected for multiple hypothesis testing using the Benjamini – Hochberg correction [34] and only results below a 5% (or lower) false discovery rate (FDR) threshold were considered significant. The Mann – Whitney U test [35] was used to assess whether distributions were significantly different. Receiver operating characteristic (ROC) curves and confidence intervals are calculated using a bootstrap procedure with 1000 iterations performed using the pROC package [36]. The riverplot package [37] was used to draw the Sankey diagram, while all other plots were generated with ggplot2 [38].

## Results

**Fig. 1:**
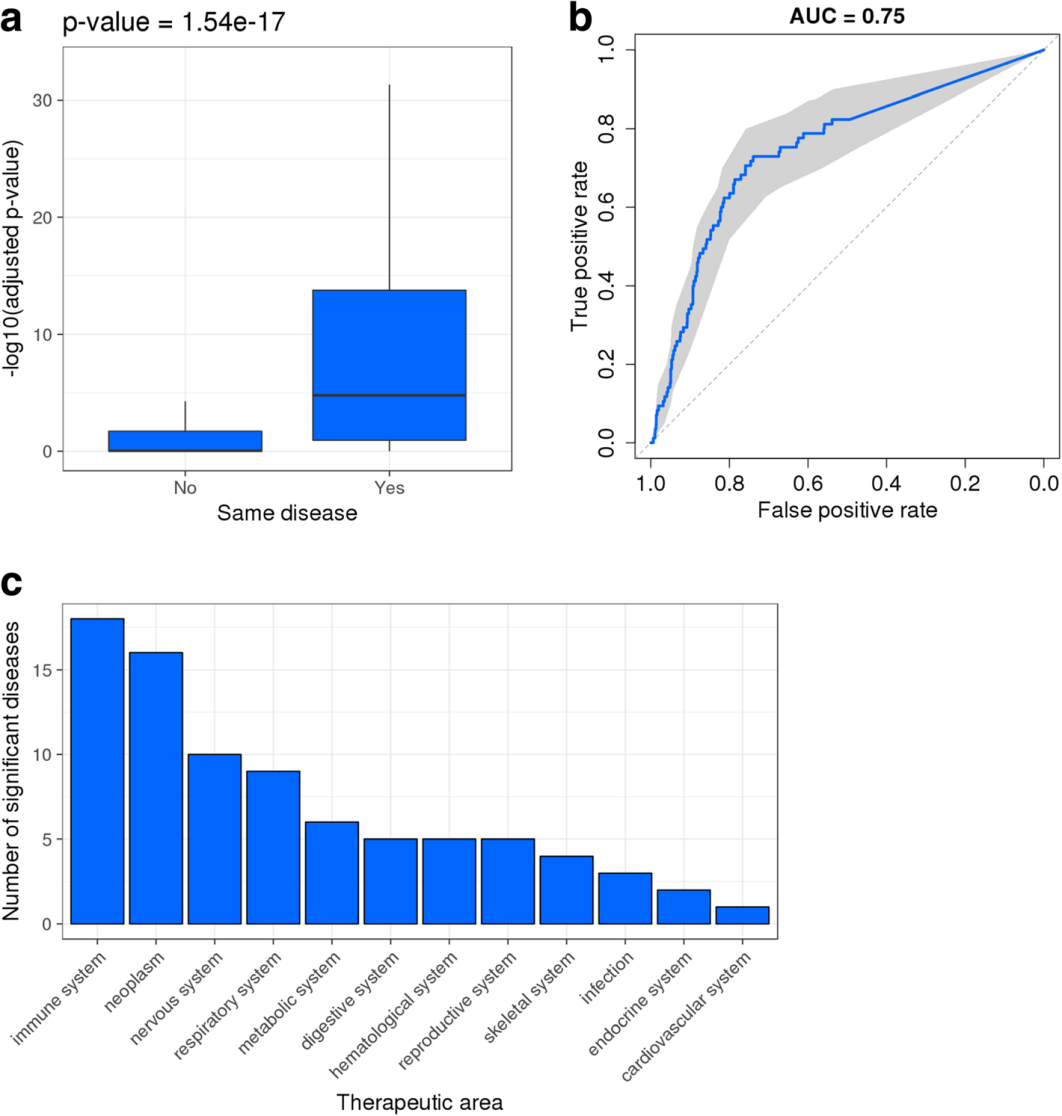
Genes differentially expressed in disease are enriched for genes genetically associated with the same disease. **a** Boxplots showing distributions of Fisher’s exact test *p*-values for genetic and transcriptomic gene sets overlaps from the same or different diseases. **b** ROC curve with 95% confidence intervals obtained using same or different disease as labels and significance of enrichment between genetic and transcriptomic gene sets as the ranking metric. **c** Barplot showing breakdown of diseases by therapeutic area for which there is a significant overlap between GWAS associations and genes differentially expressed

We set out to assess whether gene associations from GWASs could be leveraged to formulate drug repositioning hypotheses. First, we asked whether genes that are genetically associated with a disease are more likely to also be differentially expressed in that same disease, when compared to any other disease. As conventional drug repositioning approaches typically rely on transcriptomic readouts as disease representations, if there is a significant overlap between genetic associations and differentially expressed genes (DEGs) in any given disease, then we argue that GWAS data could replace or supplement transcriptional signatures in such workflows. We refer to this as Hypothesis 1: *genes differentially expressed in disease X are enriched for genes genetically associated with disease X, compared to other diseases*. For each disease, we obtained GWAS hits from STOPGAP [26] and lists of genes differentially expressed in both directions from Open Targets [27]. We then calculated the odds ratio and the significance of the overlap between gene sets for each pairwise disease combination using Fisher’s exact test. We compared the *p*-values distributions of gene sets from the same disease and from different diseases and observed a statistically significant difference (*p-*value = 1.54e-17), with gene sets from the same disease more likely to show a significant overlap between genetic and transcriptomic hits (Fig. 1a). To quantify the predictive power of our observation, we carried out a receiver operating characteristic (ROC) analysis by considering the negative base 10 logarithm of the adjusted *p*-values as the ranking metric and whether the two gene sets came from the same disease or not as labels (Fig. 1b). We observed a total area under the curve (AUC) of 0.75 (95% confidence interval [0.70, 0.80]) suggesting it is possible to predict whether a genetic and a transcriptomic gene set originate from the same disease based on the significance of their overlap. The list of diseases with significant overlap between DEGs and GWAS associations (adjusted *p-*value < 0.05) includes several immune diseases as well as a proportion of oncology, neurological and respiratory indications (Fig. 1c and Additional file 1: Table S1). These results highlight the confluence of genetic association and gene expression changes in disease and suggest that GWAS genes with dysregulated expression might be better initial candidates for drug target discovery.

**Fig. 2:**
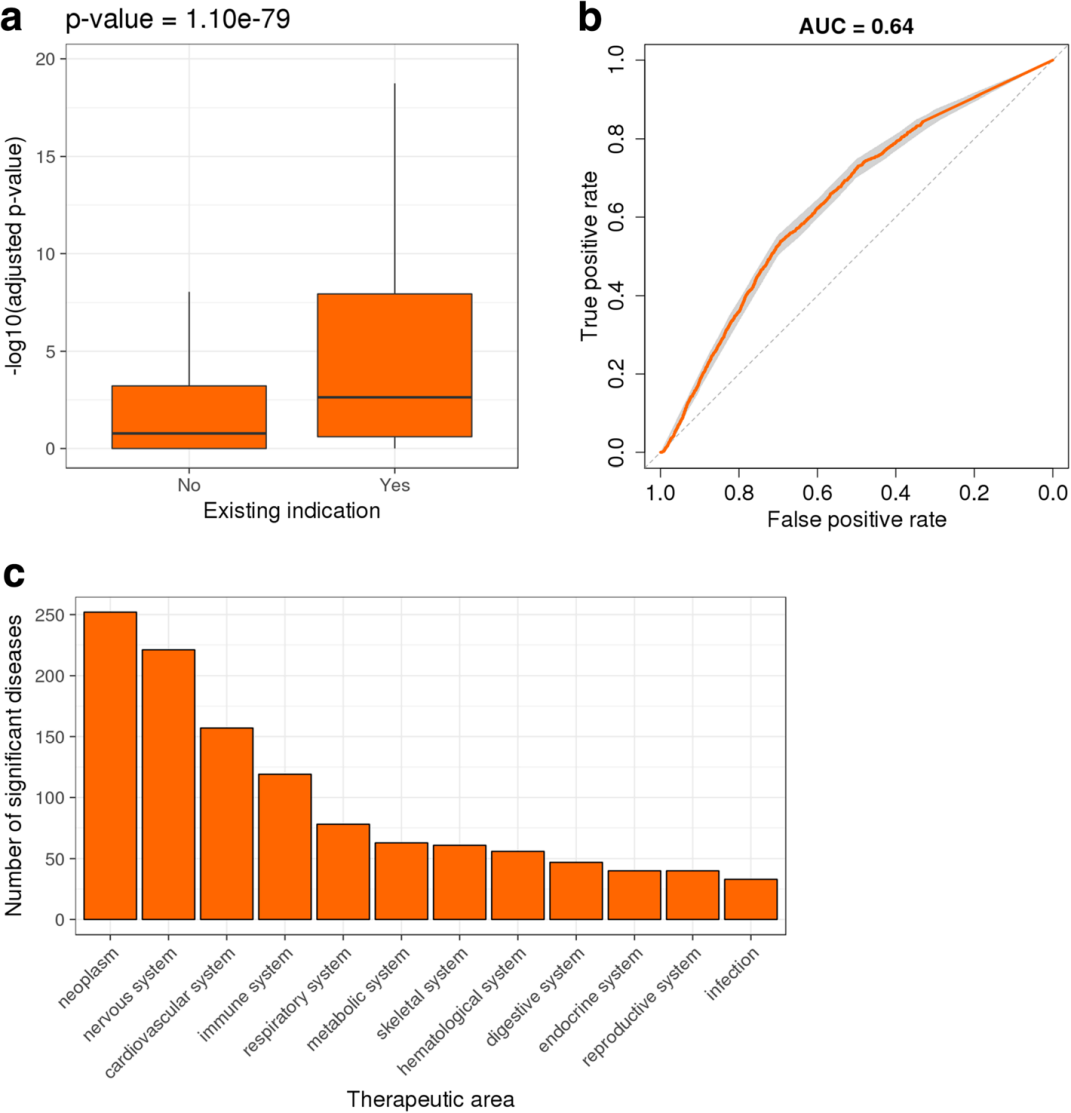
Genes differentially expressed after treatment with drug approved for a disease are enriched for genes genetically associated with the same disease. **a** Boxplots showing distributions of Fisher’s exact test *p-*values between GWAS disease associations and DEGs after drug treatment for current and other indications. **b** ROC curve with 95% confidence interval obtained using current indications as positive labels and significance of enrichment between genetic hits in disease and drug transcriptomic profiles as the ranking metric. **c** Barplot showing breakdown of current drug indications by therapeutic area where there is a significant overlap between GWAS associations and DEGs after drug treatment

We then asked whether, for indications with commercially available drugs, genes transcriptionally modulated by the drug are enriched for genes genetically associated with the disease. For indications showing enrichment, we propose that the drug causing significant expression changes in this set of GWAS genes could constitute a potential repositioning option. We refer to this as Hypothesis 2: *genes differentially expressed after treatment with drug Z for disease X are enriched for genes genetically associated with disease X*. We retrieved all 595 indications of 1555 current drugs from Open Targets [27] and calculated the significance of the overlap between genes differentially expressed after drug treatment and genes genetically associated with disease for all drug – disease combinations using Fisher’s exact test. We then split the dataset into two groups according to whether the drug was already approved for that indication and assessed whether the corresponding distributions of adjusted *p*-values were different. We found that the *p-*values of the 7351 approved drug – indication pairs were considerably lower than the rest (*p-*value = 1.10e-79, Fig. 2a). This shows that, in several cases, approves drugs do indeed regulate the expression of genes genetically associated with the disease, and suggests that drugs can be repositioned based on the overlap between the genes they modulate and the genetic hits in target diseases. We generated ROC curves by using the significance of the overlap between the two gene sets as predictions and whether the drug – disease association was an approved one or not as labels and observed an AUC of 0.64 (95% confidence interval [0.63, 0.65], Fig. 2b), highlighting that this approach can classify existing and non-existing drug – indication pairs based on the overlap between genetic hits from the disease and genes modulated by the drug. We recover 911 significant existing drug – disease associations (adjusted *p*-value < 0.05), particularly for diseases in oncology, immunology and neurological and cardiovascular therapeutic areas (Fig. 2c and Additional file 2: Table S2).

**Fig. 3:**
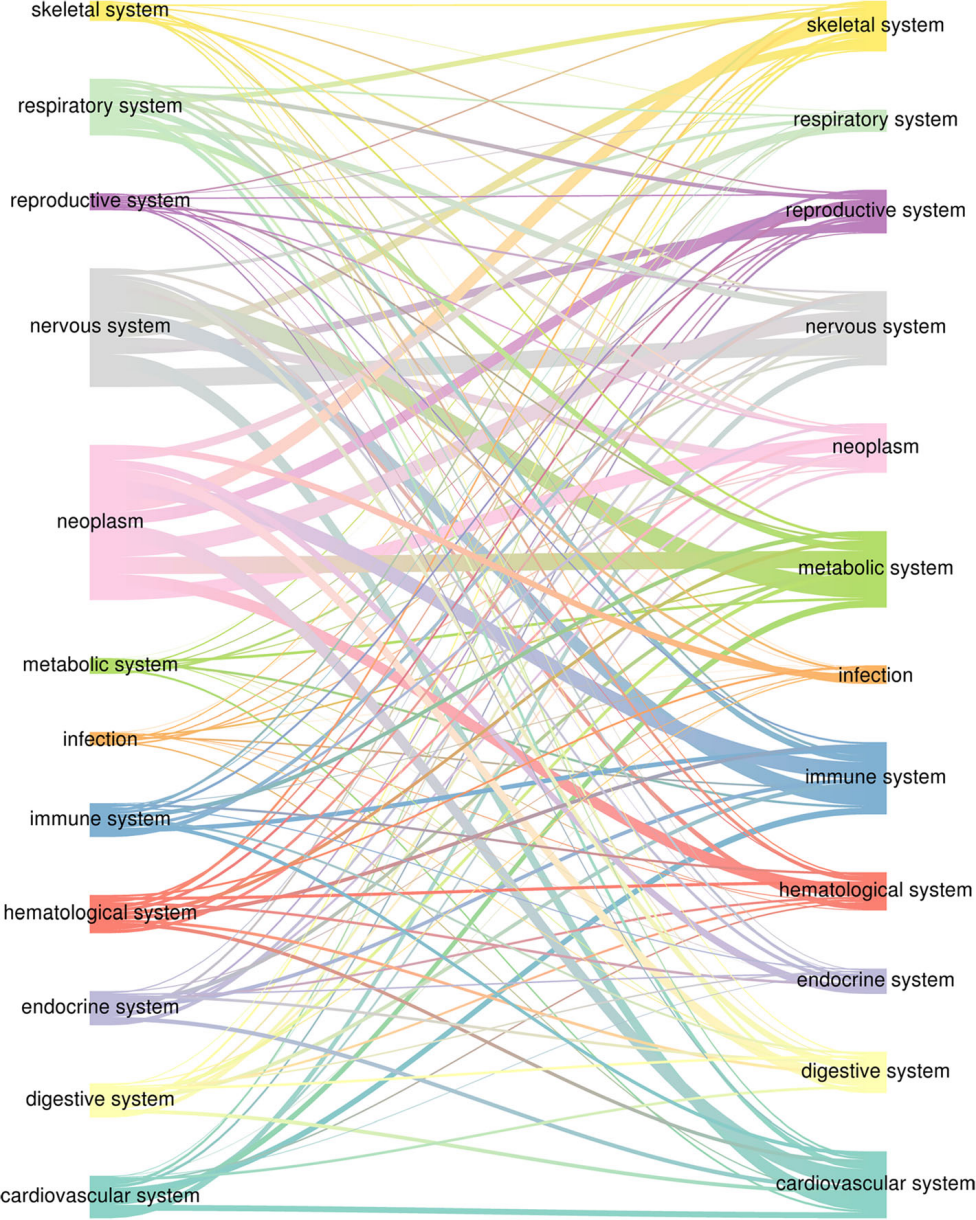
GWAS-driven drug repositioning hypotheses by therapeutic area. Sankey diagram showing all significant drug repurposing trajectories across different therapeutic areas

Overall, these results show that successful drug – disease combinations tend to display a significant overlap between the genetic background of the disease and the transcriptional response to the drug used to treat the disease. Hence, we propose to utilise the most significant, though not yet approved and perhaps not even tested, drug – indication pairs resulting from this analysis as drug repositioning hypotheses. To limit false positives, we filtered our results using a stringent adjusted *p-*value threshold (1e-10) and identified nearly 9000 such opportunities which could be prioritized and tested (Additional file 3: Table S3). Visualisation of the entire repurposing space (Fig. 3) reveals oncology and neurology as the two therapeutic areas with the largest pool of approved drugs that could be repositioned elsewhere (1093 and 926 compounds, respectively), followed by respiratory (654). However, many oncology drugs are not suitable for other indications because of their toxicity profiles. Nervous system indications could also be among the largest recipient of repurposed drugs (709), together with metabolic (719) and immune diseases (703). More specifically, the most promising trajectories appear to be nervous system to metabolic system (113 drugs), nervous system to nervous system (108), neoplasm to metabolic system (106), neoplasm to immune system (105) and nervous system to immune system (104).

## Conclusions

We showed that genes genetically associated with a disease often significantly overlap with genes differentially expressed in the same disease, as well as with genes induced or repressed by drugs used to treat that disease. To our knowledge, this is the first report to test and validate these hypotheses. We presented a simple approach to generate target prioritisation and drug repositioning hypotheses that are driven by the genetic background of the disease. Unlike more conventional repurposing approaches that rely on reverse matching of drug and disease transcriptomic signatures, we have taken advantage of the notion that genetic evidence is crucial to maximise the chances of success of drug discovery programmes [15, 16].

Our in silico framework returns a large number of statistically significant results and validation of these hypotheses would require extensive experimental work. We believe this is a major limitation of our work: we are acutely aware of the many challenges and low success rates of drug discovery programmes and recognise that a considerable proportion of our hits could be false positives. Diseases with several associated genes and drugs eliciting large transcriptional responses are more likely to result in significant results simply because of the size of these gene sets and the methodology used to compute significance.

Another issue is the lack of directionality in the genetics data we use to represent the disease space. While other Connectivity Map-inspired methods exploit up- or down-regulated genes in the transcriptomic data to identify compound profiles reversing a disease signature [7, 10, 11], our method does not take directionality into account. This could result in false positives, including predictions which could actually worsen the disease state.

In conclusion, our work represents a proof of concept that combining disease genetic and drug transcriptomic data is a valuable approach for GWAS-based drug repositioning. However, we recognise that much work remains to be done to improve its real-world applicability and would like to encourage further research in this area.

## Additional files

Additional file 1: **Table S1.** List of diseases with a significant overlap between genes differentially expressed and genes genetically associated with disease. Results are filtered for adjusted *p*-value < 0.05. (CSV 9 kb)

Additional file 2: **Table S2.** List of current drug indications with a significant overlap between genes significantly up- or down-regulated after drug treatment and GWAS hits. Results are filtered for adjusted *p-*value < 0.05. (CSV 119 kb)

Additional file 3: **Table S3.** Drug repositioning hypotheses based on the significance of the overlap between genes genetically associated with the disease and genes differentially expressed after drug treatment. Results are filtered for adjusted *p-*value <1e-10. (CSV 1089 kb)

## Abbreviations

AUC: Area under the curve
DEG: Differentially expressed gene
EFO: Experimental Factor Ontology
FDR: False discovery rate
GWAS: Genome-wide association study
LINCS: Library of Integrated Network-based Cellular Signatures
PheWAS: Phenome-wide association study
ROC: Receiver operating characteristic
